# The Brazos Valley Medical Association

**Published:** 1901-12

**Authors:** W. B. Briggs

**Affiliations:** Secretary


### The Brazos Valley Medical Association.

The Brazos Valley Medical Association was called to order by the president, Dr. Daniel Parker, at 2:30 p. m., in the city of Bryan, Texas, November 12, 1901.

Dr. F. R. Collard, first vice-president of the association, made a feeling invocation.

Dr. T. J. Pugh delivered the address of welcome on the part of the medical profession.

The address of welcome on the part of the Business League and the citizens of Bryan was made by E. A. Berry, Esq.

Dr. H. W. Cummings was called upon to respond to the addresses of welcome.

Minutes of last meeting read and adopted.

SECTION ON MATERIA MEDICA.—Report of chairman, Dr. W. S. Parker, Calvert, Texas.

PAPER: "Therapeutic Values of Water," Dr. L. M. Bassett, Hearne, Texas. This was one of the best papers ever read before the association, and was thoroughly discussed.

SECTION ON GENERAL MEDICINE.—Report of chairman, Dr. Joseph Mullen, Houston, Texas.

PAPER: "Nasal Condition as a Common Cause of Neuralgia of the Terminal Branches of the Nerves," Dr. Joseph Mullen, Houston, Texas. This paper was discussed by Drs. Daniel Parker, J. E. Thompson, Galveston Medical College; A. C. Scott, R. S. Carroll, F. R. Collard, F. E. Daniel and others.

PAPER: "The Record of Yellow Fever in the United States for the Past Does not Sustain the Mosquito Theory," Dr. J. P. Oliver, Caldwell. An abundance of discussion, pro and con, was brought forth by this most interesting paper. [See editorial.—ED.]

PAPER: "Slow Fever," Dr. E. O. Boggs, Marquez, Texas. This subject, so well handled by Dr. Boggs, was familiar to all and elicited much discussion.

SECTION ON SURGERY AND GYNECOLOGY.—Report of chairman, Dr. A. C. Scott, Temple, Texas.

PAPER: "The Surgery of the Submaxillary Region, with Special Reference to the Treatment of Cancer of the Tongue and the Floor of the Mouth," Dr. J. E. Thompson, Galveston Medical College. This grand paper was discussed by Drs. Parker, Fountain, Scott, Mullen, Carroll, Cummings and others. To make this subject better understood, Dr. Thompson brought with him a head and neck, showing a most beautiful and careful dissection of the submaxillary region, exposing the glands, jugular vein, arteries and nerves. Intense interest was manifested.

PAPER: "Intestinal Anastimosis by a Simple and Entirely New Method," Dr. A. C. Scott, Temple, Texas. Dr. Scott, after reading his most able and interesting paper, proceeded to demonstrate the operation, and received the closest attention.

PAPER: "Collegebred Women and Non-Collegebred Women," Dr. Ben H. Brodnax, Brodnax, Louisiana. A lengthy discussion followed the reading of this well written paper. (Whenever the subject of woman comes up, everybody has something to say, not even excepting the staid Dr. F. E. Daniel, of the "Red Back" journal.) Dr. Brodnax will furnish other papers.

PAPER: "What Killed McKinley?" Dr. T. J. Pugh, Bryan, Texas. Dr. Pugh thinks if McKinley had been placed in the hands of Texas physicians, it would have been better. The discussion became general and deeply interesting; so much so, that the president was compelled to limit the time.

SECTION ON OBSTETRICS AND PEDIATRICS.—Dr. F. J. Gilson, chairman, being absent, no report was made.

PAPER: "Forceps Operation in Obstetrics," Dr. A. G. Krueger, Caldwell, Texas. Dr. Krueger was highly complimented on the thoroughness of his paper.

PAPER: "Care of the Perineum During and After Labor," Dr. E. O. Boggs, Marquez, Texas. This short but finely written paper provoked a deal of discussion, and closed the list of papers.

A vote of thanks was tendered the author of each paper.

The following resolution endorsing the appointment of Dr. George R. Tabor as State Health Officer by Gov. J. D. Sayers was adopted unanimously and by a rising vote:

"WHEREAS, The Governor, the Hon. J. D. Sayers, has seen fit to appoint as State Health Officer Dr. Geo. R. Tabor, of Bryan; and,

"WHEREAS, We know Dr. Tabor to be a gentleman of integrity, honesty and ability, with especial tact and fitness for said position; and further, because Dr. Tabor is one of the founders and promoters of the Brazos Valley Medical Association, as well as an ex-president of same; be it

"*Resolved,* That we highly commend said action of the Honorable Governor Sayers, as well as feel proud that of all the good material in the State from which he could have chosen, and chosen well, a successor to former Health Officer Blunt, he saw fit to take a member and ex-officer of our association."

The following resolution relative to changing the place of meeting of the Texas State Medical Association from El Paso to some point more centrally located and within reach of the members was unanimously adopted:

"WHEREAS, The State Medical Association at its last meeting selected El Paso for its next meeting place; and,

"WHEREAS, The location of El Paso is such as to make it impracticable, if not impossible, for the average member of said Association, as well as others who might wish to do so, to attend said meeting; and,

"WHEREAS, The next meeting will be one at which material matters of interest to every member will be discussed and acted upon; and,

"WHEREAS, We believe a vast majority of said members, had opportunity to do so been permitted, would have protested against said meeting being held so far from the center of the State; therefore, be it

*"Resolved* by this association, That we urge the officers of the State Association to make an effort to have the meeting place changed to some other city more centrally located, thus insuring a better and larger attendance at same."

The following tribute of respect on the deaths of Drs. E. A. Thompson, of Navasota, and R. K. Fountain, of Jones Prairie, were adopted:

"WHEREAS, It has pleased the Creator of all mankind in his wisdom to call from our midst our professional brethren and co-laborers, Dr. E. A. Thompson, of Navasota, and Dr. R. K. Fountain, of Jones Prairie. Be it

*"Resolved,* That in the death of these two progressive and earnest members the Brazos Valley Medical Association has sustained a loss, indeed; and be it further

*"Resolved,* That these resolutions of respect and regret be entered on the minutes of this Association.

"G. W. EMORY,
"F. R. COLLARD,
"ROBT. S. CARROLL,
"Committee."

The following resolution of thanks was adopted by a rising and unanimous vote:

*"Be it resolved,* That this association express its thanks to the citizens and physicians of Bryan for their hospitality and reception of our association during its meeting in their city.

*"Be it further resolved,* That we desire to express our appreciation of Mr. M. H. James and Drs. Reed and McDougle for courtesies extended to us as well as their presence with us in our deliberations.

*"Be it further resolved,* That we highly appreciate the consideration shown us by the press of the city in publishing the proceedings of our meeting."

About forty physicians were in attendance at the Bryan meeting. The association received six new members.

Marlin was selected as the place for the next meeting, the time being the second Tuesday and Wednesday in May, next.

Drs. J. E. Thompson, of Galveston; Joseph Mullen, of Houston, and F. E. Daniel, of Austin, who live at the greatest distances, have never missed a meeting since they were made members. Those living nearer should follow their example. This was one of the best meetings ever held by the association, and continued until 10 p. m. on the 13th, inst., when it adjourned.

The banquet at the Exchange Hotel was all that could be desired—everybody happy.

W. B. BRIGGS, Secretary.

[All the papers read at this meeting will appear in the TEXAS MEDICAL JOURNAL, the official organ of the society.—ED.]

---

